# Sulphur geodynamic cycle

**DOI:** 10.1038/srep08330

**Published:** 2015-02-09

**Authors:** Takanori Kagoshima, Yuji Sano, Naoto Takahata, Teruyuki Maruoka, Tobias P. Fischer, Keiko Hattori

**Affiliations:** 1 Division of Ocean-Earth System Science, Atmosphere and Ocean Research Institute, University of Tokyo, Kashiwa, Chiba 277-8564, Japan; 2 Graduate School of Life and Environmental Sciences, University of Tsukuba, Tsukuba, Ibaraki 305-8572, Japan; 3 Department of Earth and Planetary Sciences, University of New Mexico, Albuquerque, New Mexico 87131, USA; 4 Department of Earth Sciences, Advanced Research Complex, University of Ottawa, Ottawa, ON K1N 6N5, Canada

## Abstract

Evaluation of volcanic and hydrothermal fluxes to the surface environments is important to elucidate the geochemical cycle of sulphur and the evolution of ocean chemistry. This paper presents S/^3^He ratios of vesicles in mid-ocean ridge (MOR) basalt glass together with the ratios of high-temperature hydrothermal fluids to calculate the sulphur flux of 100 Gmol/y at MOR. The S/^3^He ratios of high-temperature volcanic gases show sulphur flux of 720 Gmol/y at arc volcanoes (ARC) with a contribution from the mantle of 2.9%, which is calculated as 21 Gmol/y. The C/S flux ratio of 12 from the mantle at MOR and ARC is comparable to the C/S ratio in the surface inventory, which suggests that these elements in the surface environments originated from the upper mantle.

Volcanic and hydrothermal activity discharge sulphur and carbon from the Earth's mantle to the atmosphere and hydrosphere. Sulphur and carbon also dissolve in seawater and are incorporated into sediments before being recycled back into the mantle^1^ through subduction. The global flux of sulphur gas from sub-aerial arc volcanoes is well constrained^1^,^2^ based on measurements of SO_2_ gas from volcanoes using correlation spectrometry (COSPEC^3^), UV spectroscopy, and satellite remote sensing^2^. However the main mantle flux of volatile elements is derived from MOR-type volcanism on divergent plate boundaries of the Earth. Therefore, submarine flux must be studied together with sub-aerial flux to evaluate a mass balance of sulphur and carbon on the Earth's surface. However, the sulphur flux from submarine volcanism, which includes the amount released from the magma and that resulting from dissolution of solidified igneous rocks, is not well understood due to difficulties inherent to ocean bottom measurements.

A sulphur flux of 7.8 × 10^10^ mol/y was estimated from seawater–basalt sulphur exchange during hydrothermal alteration^4^. This value is markedly lower than the estimate of 1.64 × 10^12^ mol/y based on oceanic crust production and its sulphur content^5^. ^3^He is a useful geochemical tracer because of its primordial origin and inert behaviour, and its mantle flux has been used for calculations of other volatile fluxes. Mantle carbon flux of 2 × 10^12^ mol/y was derived from the MOR ^3^He flux and CO_2_/^3^He ratio in MOR basalt glass^6^. However, except for a very rough estimate^7^, no report in the literature has described an estimation of mantle sulphur flux at MOR as calibrated against the mantle ^3^He flux because no S/^3^He ratios in MOR basalt glasses have yet been reported.

This report describes the sulphur flux at MOR based on crushing of basalt glass, data of high-temperature submarine vent chemistry, and recent estimates of the mantle ^3^He flux^8^,^9^. Additionally, we present the amount and the origin of sulphur in arc magmas based on calculations using δ^34^S values and S/^3^He ratios. The results enable us to compare the total natural flux with anthropogenic emissions of sulphur. We also verify the global mass balance of carbon to discuss the evolution of the atmosphere.

## Results

### Vesicles

**Figure 1:**
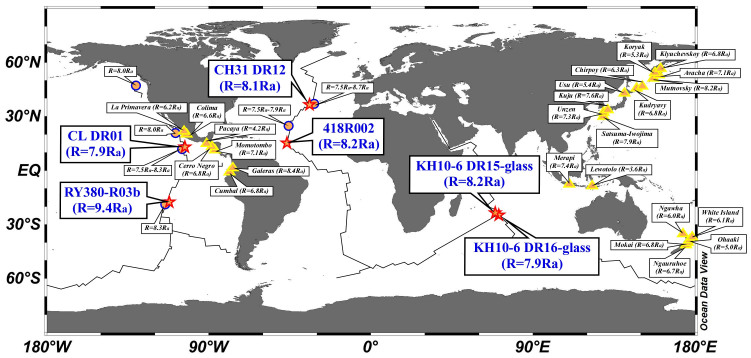
Map showing sampling sites of MOR basalt (RY380-R03b, CL DR01, 418R002, CH31 DR12, KH10-6 DR15-glass, and KH10-6 DR16-glass) analyzed in this study (stars), major hydrothermal vents (circles), and major subaerial volcanoes (triangles), together with helium isotopic signatures: *R* denotes the ^3^He/^4^He ratio in each site; *R*_a_ is the ^3^He/^4^He ratio in air of 1.382 × 10^−6^. All data are from Tables and Supplementary Tables. Lines on the ocean area show oceanic ridges. This figure was prepared using the Ocean Data View software^52^.

We analyzed MORB glass samples collected at six sites on the East Pacific Rise, Mid-Atlantic Ridge, and Central Indian Ridge (Fig. 1). The ^3^He/^4^He ratios and ^3^He contents of MOR basalt glass vesicles were 7.9 *R*_a_–9.4 *R*_a_ (where *R*_a_ is the atmospheric ratio of 1.382 × 10^−6^)^10^, and from 1.9 × 10^−15^ to 5.1 × 10^−15^ mol/g (Table 1), respectively. The values agree well with data presented in an earlier report^11^. The samples show similar total sulphur contents in vesicles with an average of 1.25 × 10^−7^ mol/g. The average value of S/^3^He ratios in vesicles was (4.2 ± 1.2) × 10^7^ (1σ). Evaluating δ^34^S values of vesicle sulphur was difficult because the amount was less than the blank contribution from the filtering system used to precipitate BaSO_4_.

### Glass matrix

The ^3^He contents of MOR basalt glass matrix are listed in Supplementary Table 1. The sulphur contents of MOR basalt glass matrix measured using a secondary ion mass spectrometer (NanoSIMS; Cameca SAS, Gennevilliers, France) listed in Supplementary Table 1 are well within the variation of sulphur contents in MOR glass obtained using a conventional method^12^. The average value of S/^3^He ratios in the glass matrix was (1.0 ± 0.2) × 10^10^ (1σ). Observed δ^34^S values are consistent with those of MOR basalt and mantle values^12^,^13^, suggesting a typical mantle sulphur signature.

## Discussion

The S/^3^He ratio and ^3^He flux at MOR are needed for sulphur flux calculations. The observed S/^3^He ratios of vesicles in MOR basalt glass are 2.0 × 10^7^–9.9 × 10^7^. These values are lower than those in the glass matrix (See Table 1 and Supplementary Table 1). This observation suggests higher solubility of sulphur than of helium in basaltic melt, which is also supported by recent laboratory experiments^14^. We consider the average vesicle S/^3^He ratio of 4.2 × 10^7^ as the minimum for MOR for our flux calculations. The other independent means to estimate the S/^3^He ratio of MOR is using the chemistry of high-temperature submarine vents. The average S/^3^He ratio is (3.4 ± 0.7) × 10^8^ (1σ) among 10 high-temperature (>200°C) hydrothermal sites worldwide (Supplementary Table 2). The δ^34^S value of H_2_S in hot vent fluids is variable^13^, but the original value before the incursion of seawater is similar to the MOR basalt values^4^,^15^. A small part of H_2_S might be generated from the reduction of seawater SO_4_ from the recharge zone^4^, although it is difficult to deconvolve the contribution quantitatively. Therefore the vent S/^3^He ratio of 3.4 × 10^8^ is expected to be the maximum estimate at MOR. We take an average of these two independent estimates (1.9 ± 1.5) × 10^8^ as the upper mantle S/^3^He ratio in the current study. The S/^3^He ratio in MOR glass matrix is higher than this upper mantle value (Supplementary Table 1), which implies that helium has degassed from the melt before it was quenched to a glass and therefore these ratios should not be used for the sulphur flux estimate.

Based on the saturation anomaly of ^3^He in deep seawater of the eastern Pacific, a value of 1070 ± 270 mol/y was calculated for the ^3^He flux from MOR^16^. A more recent estimate of the MOR ^3^He flux is 530 ± 100 mol/y derived from an ocean circulation model which also considers radiocarbons and chlorofluorocarbons^8^. This MOR ^3^He flux, when combined with the average S/^3^He ratio obtained in this study, provides the MOR sulphur flux of (1.0 ± 0.8) × 10^11^ mol/y. This mantle flux is consistent with the estimate based on seawater–basalt sulphur exchange during hydrothermal alteration^4^, but it is about an order of magnitude smaller than the value calculated from the production rate of the oceanic crust and sulphur contents therein^5^. This difference suggests that most sulphur remains in the magma and solidifies as sulphides in the MOR crust, and does not contribute to the MOR flux that discharges into the ocean. When we consider the mass balance of sulphur in global ocean water, the mantle flux of 1.0 × 10^11^ mol S/y is a second-order flux 'compared to the riverine input of 8.9 × 10^11^ mol S/y to the ocean and the output of 5.5 × 10^11^ mol S/y as sedimentary pyrite and evaporitic sulphate^17^. However, the sulphur flux from the upper mantle into the ocean represents a deep Earth contribution and is therefore distinct from riverine sulphur input that is continent-derived.

Sulphur and helium isotopic compositions are useful for investigating the origin of sulphur at arc volcanoes. The thermodynamic equilibrium between SO_2_ and H_2_S together with their δ^34^S values might provide constraints on the evolution of volcanic gases, such as an isochemical cooling path, under the assumption that the initial δ^34^S_ΣS_ value is 0‰^13^, where δ^34^S_ΣS_ denotes the total sulphur isotopic ratio of SO_2_ and H_2_S. In addition, the δ^34^S_ΣS_ values might provide information related to the origin of sulphur in ARC volcanic gases, even though they might be affected by a gas-melt separation and related fractionation processes^13^,^18^. Available data of ^3^He and total sulphur contents, and δ^34^S_ΣS_ values for high-temperature volcanic gases (>200°C) in subduction zones were compiled from the literature (Table 2). Their ^3^He/^4^He ratios are consistent with the range of subduction-type He^1^,^19^. The δ^34^S_ΣS_ values are generally positive, except for one outlier from Galeras. This similarity suggests that the sulphur signature of an ARC magma source is due to incorporation of subducted sulphate partly derived from a seawater component^18^,^20^,^21^ with high δ^34^S values. The average value of S/^3^He ratios among these high-temperature ARC gases is (6.5 ± 1.1) × 10^9^ (1σ), which is significantly higher than that of the upper mantle, suggesting enrichment of sulphur in the ARC mantle source by subduction processes.

**Figure 2:**
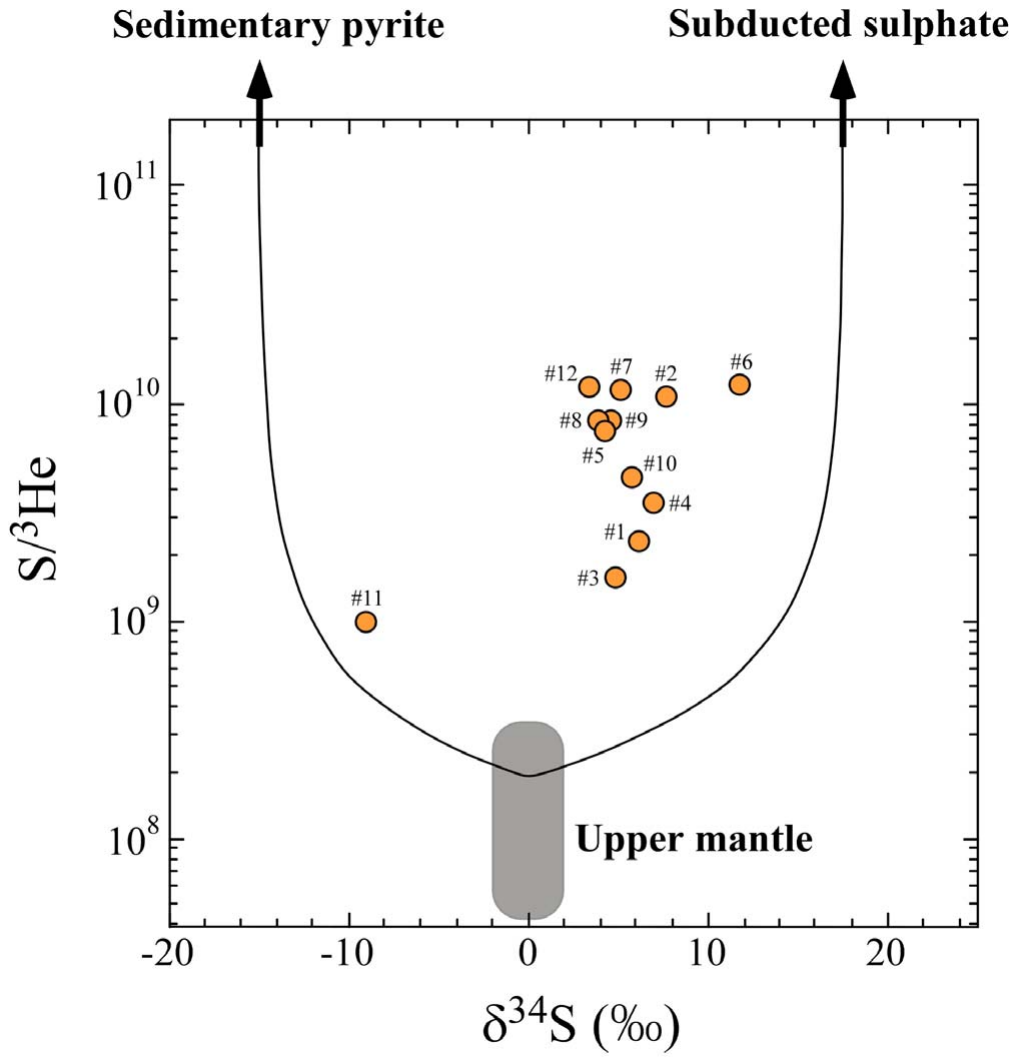
Correlation diagram between δ^34^S and S/^3^He ratios of high-temperature volcanic gases in circum-Pacific regions. Model end-members of the upper mantle, sedimentary pyrite and subducted sulphate are included. The curve shows mixing among the end-members. ^#^Data are of the following volcanoes: (#1) Avacha, (#2) Mutonovsky, (#3) Kudryavy, (#4) Usu, (#5) Kuju, (#6) Satsuma-Iwojima, (#7) Lewotolo, (#8) White Island, (#9) Ngauruhoe, (#10) Momotombo, (#11) Galeras, (#12) Colima. All data are from Table 2.

Fig. 2 presents the relation between S/^3^He ratios and δ^34^S_ΣS_ values of volcanic gases in subduction zones. The figure particularly shows end-member data for the upper mantle, sedimentary pyrite with reduced sulphur derived from slab, and subducted sulphate. The δ^34^S values of sedimentary pyrite vary considerably due to the result of bacterial reduction of seawater sulphate, and have a mean value of −20.9‰ in the Western Pacific^22^. Results of a recent study^23^ of the oceanic basement in northern Italy suggest that low-temperature serpentinization produces a negative δ^34^S_ΣS_ value with (−8.9 ± 8.0)‰. Then the δ^34^S value of sedimentary pyrite is defined as (−14.9 ± 6.0)‰. No source of primordial helium exists in the pyrite, and the slab may have lost the original mantle helium as well^24^. It is therefore possible to adopt S/^3^He larger than 1 × 10^13^ for the sedimentary pyrite. Seawater sulphate has a δ^34^S value of +21.0‰^25^. Metasomatic fluids released from sediment, of which the sulphur is mostly in the form of sulphate, have a δ^34^S value of +14‰ when their sulphur compositions resemble the bulk sediment composition^21^. Using these values, the δ^34^S value of subducted sulphate is here defined as (+17.5 ± 3.5)‰. A defined S/^3^He larger than 1 × 10^13^ for sedimentary sulphate is consistent with the seawater SO_4_/^3^He of 1.0 × 10^14^.

The distribution of volcanic gas data in the S/^3^He-δ^34^S diagram (Fig. 2) suggests that ARC samples are explained by three-component mixing. When sulphur in a sample is a mixture of the upper mantle, subducted sedimentary pyrite, and subducted sulphate having respective masses *M*, *P*, and *S*, the following equations can be derived:
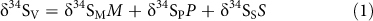

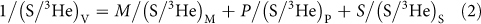
 Therein, the following relation holds:
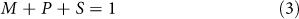
In those equations, subscripts V, M, P and S respectively denote the volcanic gas, the upper mantle, subducted sedimentary pyrite and subducted sulphate. Taking values of δ^34^S_M_ = 0‰, δ^34^S_P_ = −14.9‰, δ^34^S_S_ = +17.5‰, (S/^3^He)_M_ = 1.9 × 10^8^, (S/^3^He)_P_ = 1.0 × 10^13^ and (S/^3^He)_S_ = 1.0 × 10^13^, one can calculate the percentage of the three components *M*, *P*, and *S* quantitatively in ARC samples (Table 2). The uncertainty of estimated contributions is assessed in the Supplementary Discussion. The contribution of mantle sulphur is 1.5%–19% (2.9% average) in ARC samples, and the main contribution derives from subducted sulphate and sedimentary pyrite (See Supplementary Discussion for calculations). Volcanic gas from Satsuma-Iwojima shows the highest subducted sulphate contribution with the highest δ^34^S_ΣS_ value. To explain the heavy δ^34^S_ΣS_ values of ARC volcanic gases, incorporation of a seawater component in the magma source has been inferred since the 1970s^20^. Here we first provide a quantification of the relative amount of the seawater sulphate contribution to ARC gases, which allows us to evaluate the recycling capacity of ARC volcanoes within the global sulphur cycle.

A conventional ARC ^3^He flux was estimated from the MOR flux, given the assumption that the magma production rate of ARC is about 20% of that of MOR^9^. This percentage is consistent with the estimate of global magma emplacement and volcanic output averaged over the last 180 m.y.^26^. Recently, the MOR ^3^He flux was calculated to be 530 mol/y^8^ which would result in an ARC ^3^He flux of 110 ± 20 mol/y. This flux is consistent with the value obtained by summation of ^3^He flux at arc volcanoes worldwide^1^. The average S/^3^He ratio of (6.5 ± 1.1) × 10^9^ (1σ) is obtained from high-temperature volcanic gases. Therefore, the ARC sulphur flux is estimated to be (7.2 ± 1.8) × 10^11^ mol/y based on the ^3^He flux of 110 mol/y. This value is considerably larger than MOR sulphur flux calculated in this study. However the upper mantle contribution to ARC volcanic gases is only (2.9 ± 0.5)% of total sulphur, on average. The sulphur flux from the wedge mantle at ARC then becomes (2.1 ± 0.6) × 10^10^ mol/y, which is less than the mantle sulphur flux discharging into the ocean at MOR. The major contribution of the ARC sulphur flux is derived from subducted sedimentary pyrite and subducted sulphate partly derived from the seawater component.

**Figure 3:**
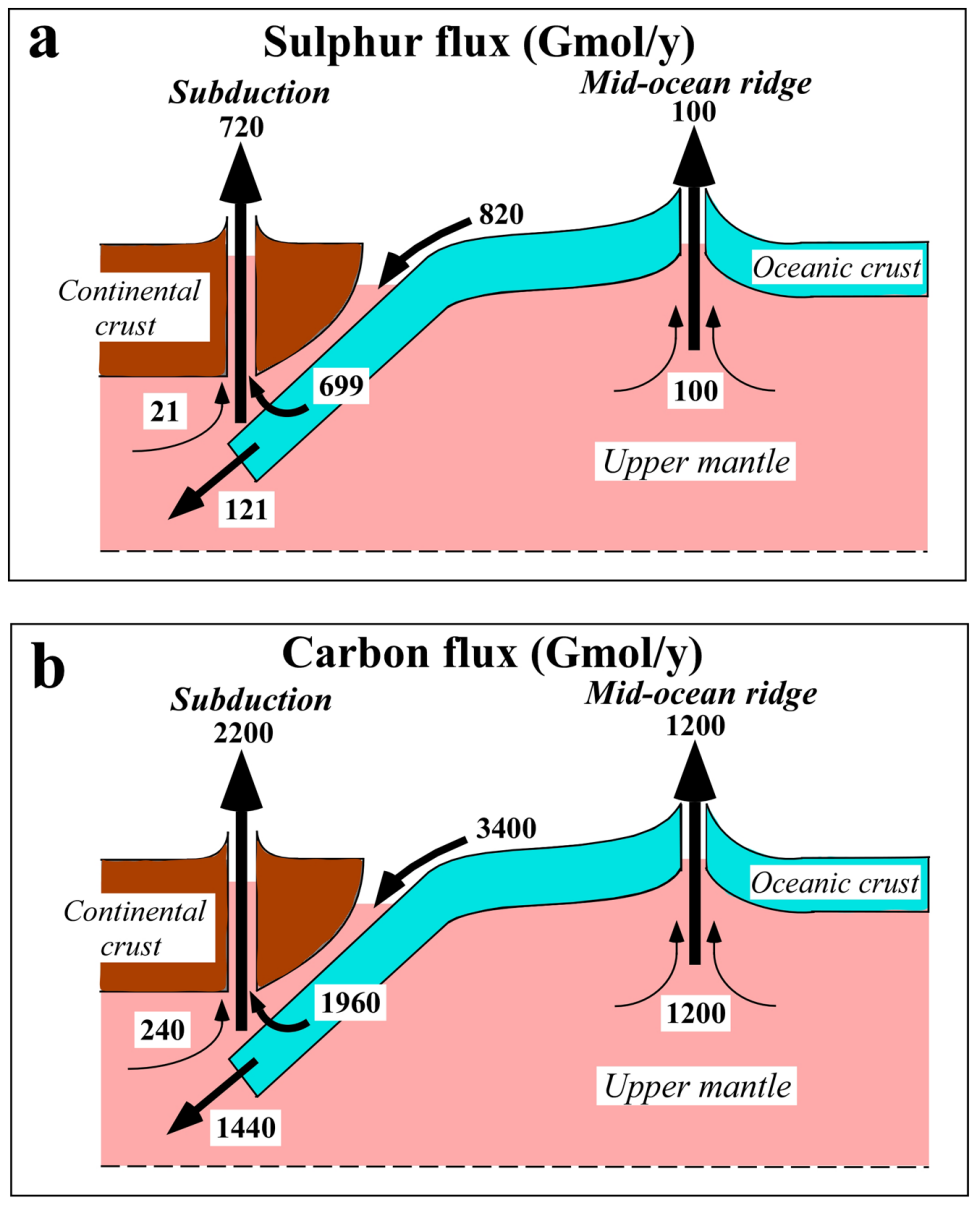
Schematic diagrams of (a) the global sulphur cycle and (b) the global carbon cycle. Each flux is given in units of 10^9^ mol/y. It should be noted that steady-state surface environments of these elements were applied.

A summary of the global sulphur flux is depicted in Fig. 3a. Present hot spot magmatism likely does not contribute substantially to the global flux of sulphur (See Supplementary Discussion). The total volcanic flux of sulphur is estimated as 8.2 × 10^11^ mol/y and represents about one-third of the anthropogenic emissions due to coal burning and sulphide ore smelting^27^. This natural flux, if it has remained constant over 4.55 billion years of geological time, engenders an accumulation of 3.7 × 10^21^ mol. This value is greater than the surface inventory of 5.3 × 10^20^ mol^1^. If we take the MOR flux together only with the mantle wedge flux of 2.1 × 10^10^ mol/y, then the accumulation becomes 5.6 × 10^20^ mol in total, which is equivalent to the surface inventory. When steady-state recycling of sulphur is applied, the total subducting flux becomes 8.2 × 10^11^ mol/y.

As new ^3^He flux data at MOR have been reported^8^, we revise the carbon geodynamics along with sulphur. The CO_2_/^3^He ratio at MOR was calculated to be (2.2 ± 0.7) × 10^9^ using CO_2_/^3^He data for MOR basalt glass and hydrothermal fluids^28^. This ratio, combined with the new MOR ^3^He flux, engenders the global MOR CO_2_ flux of (1.2 ± 0.4) × 10^12^ mol/y, which is consistent with the most recent estimate based on vesicularities of MORB worldwide^29^.

**Figure 4:**
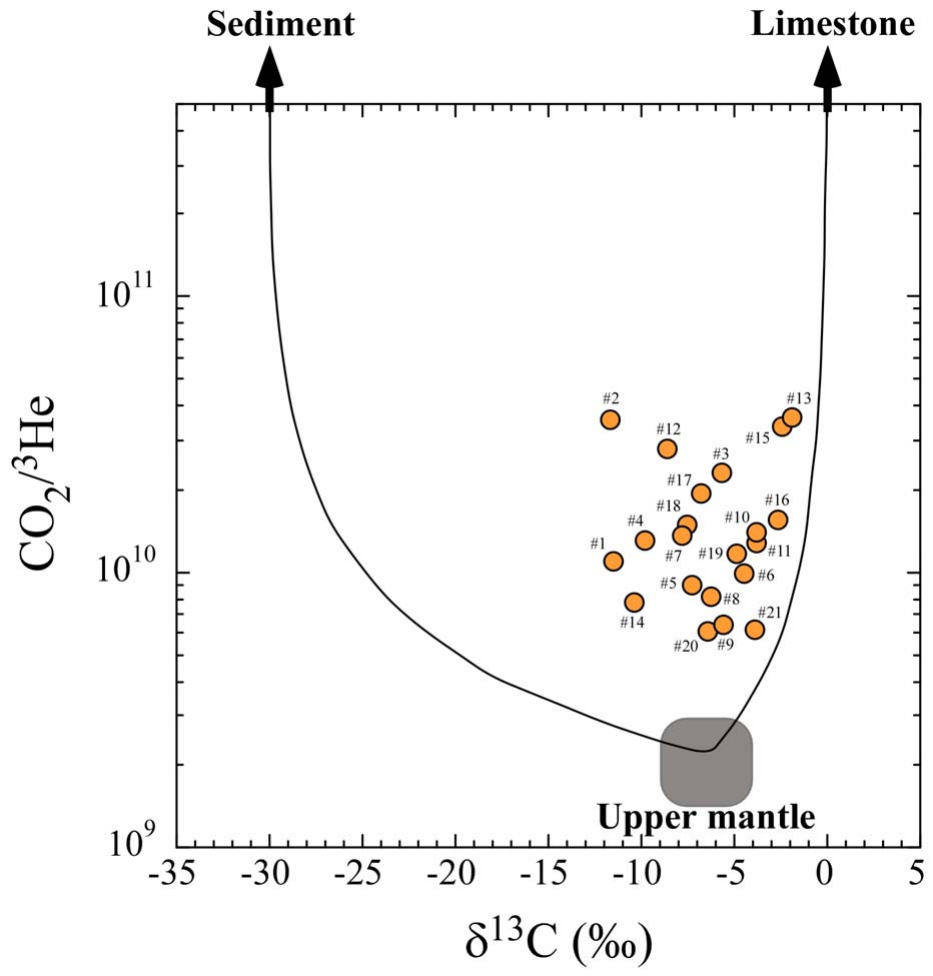
Correlation diagram between δ^13^C and CO_2_/^3^He ratios of high-temperature volcanic gases in circum-Pacific regions. Model end-members of the upper mantle, sediment and limestone are included. The curve shows mixing among the end-members. ^#^Data are those of the following volcanoes: (#1) Klyuchevskoy, (#2) Koryak, (#3) Avacha, (#4) Mutnovsky, (#5) Kudryavy, (#6) Usu, (#7) Kuju, (#8) Unzen, (#9) Satsuma-Iwojima, (#10) Merapi, (#11) Lewotolo, (#12) Ngawha, (#13) White Island, (#14) Ngauruhoe, (#15) Cerro Negro, (#16) Momotombo, (#17) Pacaya, (#18) Galeras, (#19) Cumbal, (#20) Colima, (#21) La Primavera. All data are from Supplementary Table 3.

For ARC volcanism, we selected 24 volcanic gas and steam well data with temperatures higher than 200°C (Supplementary Table 3). Their carbon source is well explained by the mixing of three components: The upper mantle (M), organic sediment (S) and limestone with a slab component (L) (Fig. 4; Ref. 30). These end-member components are described in Supplementary Table 3. Using those values, we calculate the respective percentages of the three components in the ARC samples (Supplementary Table 3). The contribution of the upper mantle carbon is 3.2%–36% (average 11%), whereas a major part is attributable to subducted carbonate and organic carbon. Because the average CO_2_/^3^He ratio of these data is (2.0 ± 0.3) × 10^10^, the carbon flux from ARC is (2.2 ± 0.5) × 10^12^ mol/y using the ARC ^3^He flux of 110 ± 20 mol/y, which is also consistent with the recent estimate using volcanic gas observations worldwide^31^.

A summary of global carbon flux is depicted in Fig. 3b. The total volcanic flux of carbon is 3.4 × 10^12^ mol/y, which is two orders of magnitude smaller than anthropogenic emission by fossil fuel combustion and cement production^32^. The MOR flux combined with the wedge mantle flux is 1.4 × 10^12^ mol/y. This value, if accumulated for 4.55 billion years, results in 6.6 × 10^21^ mol of carbon, which closely approximates the surface inventory of 7.0 × 10^21^ mol^1^. If steady-state recycling of carbon is applied, then the total subduction flux becomes 3.4 × 10^12^ mol/y. This estimate is consistent with the influx of carbon^1^.

In conclusion, the best estimates of MOR sulphur and carbon flux are 1.0 × 10^11^ mol/y and 1.2 × 10^12^ mol/y, respectively at present, which are less than their volcanic fluxes at ARC. Sulphur and carbon fluxes from only the mantle wedge to the surface environment at ARC are calculated as 2.1 × 10^10^ mol/y and 2.4 × 10^11^ mol/y, respectively. These data provide a C/S flux ratio of 12 which is similar to the C/S ratio in the surface inventory of 13 (Ref. 1). Our results suggest that the main source of sulphur and carbon is the upper mantle. To balance the mass between the crust and the mantle, the sulphur subducted into the mantle and not immediately recycled to the surface is expected to be equivalent to 1.2 × 10^11^ mol/y, which is about 17% of the recycling sulphur of 7.0 × 10^11^ mol/y. We calculated sulphur and carbon fluxes from the mantle based on the plausible S/^3^He and C/^3^He ratios and the recently reported ^3^He flux at MOR, which constrained geochemical cycles of sulphur and carbon, and evolutionary histories of the atmosphere and hydrosphere.

## Methods

### Glass vesicle

It is difficult to measure the abundance of sulphur species such as H_2_S and SO_2_ in vesicles of MOR basalt glass together with ^3^He because the gases are highly reactive. They easily adhere to the inner surface of a vacuum crushing vessel. We have developed a gas-extraction method, ‘Frozen Crushing Method’, by which sulphur gases are fixed immediately in semi-frozen alkaline solution during mechanical fracturing of glass^7^. The abundance of helium and ^3^He/^4^He ratios were measured using a noble gas mass spectrometer (VG5400; Waters Corp.) at the Atmosphere and Ocean Research Institute (AORI). Subsequently, the vacuum was broken and the S-bearing solution was filtered. All sulphur compounds were converted into sulphate ion by oxidation with hydrogen peroxide. The concentration was measured using an ion chromatography system (ICS-2100; Thermo Fisher Scientific Inc.) at AORI. Blank contributions of sulphur and helium were considerably smaller than the actual amounts in samples. Experimental details are presented in an earlier report^7^. Sulphate ion in the alkaline solution was converted into BaSO_4_ precipitations by adding BaCl_2_ solution and δ^34^S values obtained with an elemental analyzer (vario PYRO cube; Elementar Analysensysteme, GmbH) coupled to an isotope-ratio mass spectrometer (Delta XP; Thermo Fisher Scientific Inc.) via an interface (ConFlo IV; Thermo Fisher Scientific Inc.) at the University of Ottawa.

### Glass matrix

Sulphur contents in glass matrix were measured (NanoSIMS; Cameca SAS, Gennevilliers, France) at AORI, whereas δ^34^S values were obtained using an elemental analyzer isotope-ratio mass spectrometer system^33^ (Isoprime-EA; Isoprime Ltd.) at the University of Tsukuba.

## Author Contributions

T.K. was responsible for the research and the analyses of MOR basalt vesicles with Frozen Crushing Method. Y.S. supervised the research, and made important contributions in producing the manuscript: data compilation of hydrothermal vent and volcanic gas chemistry, figure and table preparation, and discussion of volatile cycles. N.T. is a manager of VG-5400 and NanoSIMS laboratories, and supported data interpretation. T.M. analyzed samples using Isoprime-EA and interpreted data. T.P.F. made important comments on compiled data of arc volcanic gases and volatile flux. K.H. made important comments on sulphur chemistry and discussion of volatile cycles. T.K. and Y.S. wrote the manuscript based on other authors' comments.

## Supplementary Material

Supplementary Information Supplementary materials

## Figures and Tables

**Table 1:** He isotopic compositions, S concentrations and S/^3^He ratios in MORB glass vesicles

Sample name	Number of measurements	^3^He/^4^He (R_a_)	^3^He concentration (10^−15^ mol/g)	S concentration (10^−9^ mol/g)	S/^3^He (10^6^)
(East Pacific Rise basalt)					
RY380-R03b	3	9.4 ± 0.4	2.3 ± 0.1	44 ± 13	20 ± 6
CL DR01	2	7.9 ± 0.4	3.4 ± 1.1	106 ± 24	35 ± 18
(Mid-Atlantic Ridge basalt)					
418R002	3	8.2 ± 0.3	5.1 ± 0.6	182 ± 34	36 ± 8
CH31 DR12	1	8.1 ± 0.3	1.9 ± 0.1	188 ± 12	99 ± 8
(Central Indian Ridge basalt)					
KH10-6 DR15-glass	2	8.2 ± 0.2	4.3 ± 0.2	172 ± 34	40 ± 9
KH10-6 DR16-glass	3	7.9 ± 0.2	2.7 ± 0.2	58 ± 6	22 ± 2
**Average**		**8.3 ± 0.2**	**3.3 ± 0.5**	**125 ± 26**	**42 ± 12**

Uncertainty: 1σ.

**Table 2:** He isotopic ratios, δ^34^S values and S/^3^He ratios in high temperature volcanic gases

Volcano	Location	Temperature (°C)	^3^He/^4^He (R_a_)	δ^34^S (‰)	S/^3^He (10^9^)	Upper mantle	Sedimentary pyrite	Subducted sulphate	Reference
Koryak	Kamchatka	215	5.3		8.0	2.4%			[34]
Avacha	Kamchatka	473	7.1	6.4	2.3	8.5%	29.8%	61.7%	[34,35]
Mutnovsky	Kamchatka	543	8.2	7.7	10.8	1.8%	29.3%	68.9%	[34,36]
Kudryavy	Kuril	912	6.8	4.7	1.6	12.0%	33.0%	55.0%	[34,37]
Usu	Japan	750	5.4	7.0	3.4	5.7%	29.3%	65.0%	[31,38,39]
Kuju	Japan	580	7.6	4.2	7.8	2.5%	39.7%	57.9%	[40,41,42]
Unzen	Japan	818	7.3		2.1	9.2%			[31,43]
Satsuma-Iwojima	Japan	885	7.9	11.7	12.6	1.5%	17.1%	81.4%	[34,39]
Merapi	Indonesia	803	7.4		3.1	6.1%			[34]
Lewotolo	Indonesia	490	3.6	5.2	11.6	1.6%	37.1%	61.3%	[44,45]
White Island	New Zealand	495	6.1	4.0	8.4	2.3%	40.4%	57.3%	[46,47]
Ngauruhoe	New Zealand	640	6.7	4.4	8.4	2.3%	39.3%	58.4%	[31,47,49]
Momotombo	Nicaragua	747	7.1	5.9	4.6	4.2%	33.4%	62.4%	[34,50]
Galeras	Colombia	642	8.4	-8.9	1.0	18.9%	71.3%	9.8%	[13,31,43]
Colima	Mexico	814	6.6	3.4	12.2	1.6%	42.6%	55.9%	[34,51]
**Average**			**6.8**	**4.6**	**6.5**	**2.9%**	**38.1%**	**59.0%**	
