# Supplementary material for: Sulphur geodynamic cycle
Source: Sci Rep. 2015 Feb 9;5:8330. doi: 10.1038/srep08330 (PMC4321164; doi:10.1038/srep08330)
Supplement: Supplementary Information — Supplementary materials [file srep08330-s1.pdf]

Supplementary Information

Sulphur geodynamic cycle

Takanori Kagoshima[1,*], Yuji Sano[1], Naoto Takahata[1], Teruyuki Maruoka[2], Tobias P. Fischer[3], Keiko Hattori[4]

[1]Division of Ocean-Earth System Science, Atmosphere and Ocean Research Institute, University of Tokyo, Kashiwa, Chiba 277-8564, Japan

[2]Graduate School of Life and Environmental Sciences, University of Tsukuba, Tsukuba, Ibaraki 305-8572, Japan

[3]Department of Earth and Planetary Sciences, University of New Mexico, Albuquerque, New Mexico 87131, USA

[4]Advanced Research Complex, University of Ottawa, Ottawa, ON K1N 6N5, Canada

**To whom correspondence should be addressed. E-mail: kagoshima@aori.u-tokyo.ac.jp*

Supplementary Discussion

Calculation of contribution to sulphur in ARC volcanic gas from the upper mantle, sedimentary pyrite and subducted sulphate

Uncertainty of the contribution to volcanic gas compositions from the upper mantle was examined. $S/^{3}He$ ratios of sedimentary pyrite and subducted sulphate are assumed to be

1×10^{13} for the current study. Although this value might differ considerably, the results are likely to remain unaffected. Assuming either $(S/^3He)_P = 1\times10^{12}$ and $(S/^3He)_S = 1\times10^{12}$, or $(S/^3He)_P = 1\times10^{14}$ and $(S/^3He)_S = 1\times10^{14}$, the change of the mantle fraction is less than 0.1%. In contrast, the uncertainty of the $(S/^3He)_M$ ratio bears important consequences for the estimated mantle fraction. If one takes a $(S/^3He)_M$ ratio of 4.2×10^7, which is the lowest estimate, then the estimated mantle fraction becomes about one-fifth. If one chooses 3.2×10^8, then the fraction is expected to be double. Variation of the $\delta^{34}S_P$ value from -21‰ to -9‰ engenders uncertainty of up to 20% for the estimated sedimentary pyrite fraction. That of $\delta^{34}Ss$ value from +14‰ to +21‰ engenders the uncertainty of up to 10% for the estimated subducted sulphate fraction. The variation of $\delta^{34}S_M$ from -2‰ to +2‰ causes little change of the value of the estimated mantle fraction.

The average mantle contribution was calculated based on the average $S/^3He$ ratio ($(S/^3He)_V$) of 6.5×10^9 among the 15 volcanic gases presented in Table 2 and the following equation in the text.

$$1/(S/^3He)_V = M/(S/^3He)_M + P/(S/^3He)_P + S/(S/^3He)_S \qquad \text{(S1)}$$

Therein, $(S/^3He)_M=1.9\times10^8$, $(S/^3He)_P=1\times10^{13}$ and $(S/^3He)_S=1\times10^{13}$.

Contribution to the global flux of sulphur from present hot spot magmatism

Intra-plate volcanism such as that at Hawaii or Yellowstone might release large amounts of sulphur, but no study of that phenomenon has been reported in the literature. Global hot spot ^3He flux of 2.5 mol/y was reported based on Hawaiian magma (Torgersen, 1989), which was not scaled to MOR flux as for ARC. It is not necessary to revise the value according to the new MOR flux. The $S/^3He$ ratio of Kilauea volcanic gas is 5.6×10^8, where

the S/He ratio and $^3He/^4He$ ratio were referred from the literature (Craig & Lupton, 1976; Fischer, 2008). Yellowstone and Iceland fluid samples (Hearn et al., 1990; Sano et al., 1985) show ratios of 5.0×10^7 and 1.1×10^8, which are both smaller than the Hawaiian value, although their temperatures are lower than 200°C. Then the global sulphur flux at hot spot regions might not exceed the value of 1.4×10^9 mol/y, which is almost two orders of magnitude smaller than the MOR flux of 1.0×10^{11} mol/y. This fact suggests that hot spot magmatism does not strongly affect the global sulphur cycle.

Supplementary References

Bluth, G.J. & Ohmoto, H. Sulfide–sulfate chimneys on the East Pacific Rise, 11° and 13°N latitudes. Part II: Sulfur isotopes. *Can. Mineral.* **26**, 505–515 (1988).

Bowers, T.S. et al. Chemical controls on the composition of vent fluids at 13°N–11°N and 21°N, East Pacific Rise. *J. Geophys. Res*. **93**, 4522–4536 (1988).

Butterfield, D.A. et al. Gradients in the composition of hydrothermal fluids from the Endeavour segment vent field: Phase separation and brine loss. *J. Geophys. Res.* **99**, 9561–9583 (1994).

Campbell, A.C. et al. A time series of vent fluid compositions from 21°N, East Pacific Rise (1979, 1981, 1985), and the Guaymas Basin, Gulf of California (1982, 1985). *J. Geophys. Res.* **93**, 4537–4549 (1988a).

Campbell, A.C. et al. Chemistry of hot springs on the Mid-Atlantic Ridge. *Nature* **335**, 514–519 (1988b).

Charlou, J.L. et al. Compared geochemical signatures and the evolution of Menez Gwen

(37°50′N) and Lucky Strike (37°17′N) hydrothermal fluids, south of the Azores Triple Junction on the Mid-Atlantic Ridge. *Chem. Geol.* **171**, 49–75 (2000).

Charlou, J.L., Donval, J.P., Fouquet, Y., Jean-Baptiste, P. & Holm, N. Geochemistry of high H_2 and CH_4 vent fluids issuing from ultramafic rocks at the Rainbow hydrothermal field (36°14′N, MAR). *Chem. Geol.* **191**, 345–359 (2002).

Charlou, J.L., Donval, J.P., Jean-Baptiste, P., Dapoigny, A. & Rona, P.A. Gases and helium isotopes in high temperature solutions sampled before and after ODP Leg 158 drilling at TAG hydrothermal field (26°N, MAR). *Geophys. Res. Lett*. **23**, 3491–3494 (1996).

Chiba, H., Masuda, H., Lee, S.-Y. & Fujioka, K. Chemistry of hydrothermal fluids at the TAG active mound, MAR 26°N, in 1998. *Geophys. Res. Lett*. **28**, 2919–2922 (2001).

Craig, H. & Lupton, J.E. Primordial neon, helium and hydrogen in oceanic basalts. *Earth Planet. Sci. Lett*. **31**, 369–385 (1976).

Fischer, T.P., Giggenbach, W.F., Sano, Y. & Williams, S.N. Fluxes and sources of volatiles discharged from Kudryavy, a subduction zone volcano, Kurile Islands. *Earth Planet. Sci. Lett.* **160**, 81–96 (1998).

Gerlach, T.M., Present-day CO_2 emissions from volcanos. *Eos, Trans. Am. Geophys. Union* **72**, 249-255 (1991).

Hearn, E.H., Kennedy, B.M. & Truesdell, A.H. Coupled variations in helium isotopes and fluid chemistry: Shoshone Geyser Basin, Yellowstone National Park. *Geochim. Cosmochim. Acta* **54**, 3103–3113 (1990).

Hoefs, J. *Stable Isotope Geochemistry.* 208 pp (Springer, 1980).

Javoy, M. Pineau, F. & Delorme, H. Carbon and nitrogen isotopes in the mantle. *Chem. Geol.* **57**, 41–62 (1986).

Jean-Baptiste, P. et al. Helium and oxygen isotope analyses of hydrothermal fluids from the East Pacific Rise between 17°S and 19°S. *Geo-Mar. Lett.* **17**, 213–219 (1997).

Jean-Baptiste, P., Fourré, E., Charlou, J.L., German, C.R. & Radford-Knoery, J. Helium isotopes at the Rainbow hydrothermal site (Mid-Atlantic Ridge, 36°14′N). *Earth Planet. Sci. Lett.* **221**, 325–335 (2004).

Kim, K-R., Welhan, J.A. & Craig, H. The hydrothermal vent fields at 13°N and 11°N on the East Pacific Rise: ALVIN 1984 results. *EOS*, **65**, 973 (abstract, 1984).

Marty, B. & Jambon, A. C/^{3}He in volatile fluxes from the solid Earth: implications for carbon geodynamics. *Earth Planet. Sci. Lett*. **83**, 16–26 (1987).

Marty, B. & Tolstikhin, I.N. CO_2 fluxes from mid-ocean ridges, arcs and plumes. *Chem. Geol*. **145**, 233–248 (1998).

Merlivat, L., Pineau, F. & Javoy, M. Hydrothermal vent waters at 13°N on the East Pacific Rise: Isotopic composition and gas concentration. *Earth Planet. Sci. Lett.* **84**, 100–108 (1987).

Lyon, G.L. & Hulston, J.R. Carbon and hydrogen isotope compositions of New Zealand geothermal gases. *Geochim. Cosmochim. Acta* **48**, 1161–1171 (1983).

Rudnicki, M.D. & Elderfield, H. Helium, radon and manganese at the TAG and Snakepit hydrothermal vent fields, 26° and 23°N, Mid-Atlantic Ridge. *Earth Planet. Sci. Lett.* **113**, 307–321 (1992).

Sano, Y., Urabe, A., Wakita, H., Chiba, H. & Sakai, H. Chemical and isotopic composition of gases in geothermal fluids in Iceland. *Geochem. J.* **19**, 135–148 (1985).

Shanks III, W.C. Stable isotopes in seafloor hydrothermal systems: Vent fluids, hydrothermal deposits, hydrothermal alteration, and microbial processes. In: *Stable Isotope Geochemistry. Reviews in Mineraly and Geochemistry 43* (eds Valley, J.W. &

Cole, D.R.) 469–525 (Mineralogical Society of America, 2001).

Taran, Y., Inguaggiato, S., Varley, N., Capasso, G. & Favara, R. Helium and carbon isotopes in thermal waters of the Jalisco block, Mexico. *Geofis. Int.* **41**, 459–466 (2002).

Taran, Y.A., Rozhkov, A.M., Serafimova, E.K. & Esikov, A.D. Chemical and isotopic composition of magmatic gases from the 1988 eruption of Klyuchevskoy volcano, Kamchatka. *J. Volcanol. Geotherm. Res.* **46**, 255–263 (1991).

Welhan, J.A. & Craig, H. Methane, hydrogen and helium in hydrothermal fluids at 21°N on the East Pacific Rise. In: *Hydrothermal Processes at Seafloor Spreading Centers* (eds Rona, P.A., Boström, K., Laubier, L. & Smith Jr., K.L.) 391–409 (Plenum Press, 1983).

Woodruff, L.G. & Shanks III, W.C. Sulfur isotope study of chimney minerals and vent fluids from 21°N, East Pacific Rise: Hydrothermal sulfur sources and disequilibrium sulfate reduction. *J. Geophys. Res.* **93**, 4562–4572 (1988).

Supplementary Table 1. ^{3}He and S concentrations, S/^{3}He ratios and $\delta^{34}S$ values of MORB glass matrix

Sample name	^{3}He concentration (10^{-15}mol/g)	Number of SIMS analyses	S concentration (10^{-6}mol/g)	S/^{3}He (10^{9})	Number of IRMS analyses	$\delta^{34}S$ (‰)
(East Pacific Rise Basalt)						
RY380-R03b	5.7*	18	43	7.6	3	0.45
CL DR01	4.9**	15	39	8.1	1	1.26
(Mid-Atlantic Ridge Basalt)						
418R002	1.9*	18	34	18.1	3	0.53
CH31 DR12	2.7**	15	33	12.4	1	0.78
(Central Indian Ridge Basalt)						
KH10-6 DR15-glass	6.2**	10	26	4.2	1	-0.19
KH10-6 DR16-glass	3.9**	10	38	9.9	1	0.11
**Average**	**4.2**		**36**	**10.1**		**0.49**

*: Referred from Kagoshima et al. (2012)

**: Estimated based on vesicle ^{3}He contents and glass/vesicle abundance ratios of ^{3}He in RY380-R03b and 418R002

Supplementary Table 1
Kagoshima et al. (2015)

Supplemenatry Table 2. He isotopic compositions, H_2S concentrations and $\delta^{34}S$ values of H_2S in hydrothermal fluids from mid-ocean ridges

Hydrothermal site	Temperature (°C)	$^3He/^4He$ (R_a)	^3He p M	H_2S m M	$d^{34}S$ (‰)	$S/^3He$ (10^7)	Reference
(Eastern Pacific)							
Juan de Fuca (47-48°N)	370	8.0	17.4	4.4		25.3	Butterfield et al. (1994)
East Pacific Rise (21°N)	340	7.8	17.3	7.5	3.1	43.4	Woodruff & Shanks (1988); Welhan & Craig (1983); Kim et al. (1984); Campbell et al. (1988a)
East Pacific Rise (13°N)	380	7.5	24.7	5.2		21.0	Kim et al. (1984); Merlivat et al. (1987); Bowers et al. (1988)
East Pacific Rise (11°N)	347	8.3	18.2	8.2	4.7	45.1	Kim et al. (1984); Bowers et al. (1988); Bluth & Ohmoto (1988)
East Pacific Rise (17-19°S)	382	8.3	9.2	7.0	6.2	75.4	Shanks (2001); Jean-Baptiste et al. (1997)
(Mid-Atlantic Ridge)							
Menez Gwen (38°N)	284	8.7	17.6	1.6		9.1	Charlou et al. (2000)
Lucky Strike (37°N)	324	8.1	4.5	2.7		61.0	Charlou et al. (2000)
Rainbow (36°N)	365	7.5	25.0	1.2		4.8	Charlou et al. (2002); Jean-Baptiste et al. (2004)
TAG (26°N)	358	7.5	15.8	4.0	8.5	25.4	Charlou et al. (1996); Rudnicki & Elderfield (1992); Chiba et al. (2001)
Snake Pit (23°N) MARK	350	7.9	19.4	5.9	5.0	30.5	Rudnicki & Elderfield (1992); Campbell et al. (1988b)
**Average**		**8.0**	**16.9**	**4.8**	**5.5**	**34.1**	

Supplementary Table 2
Kagoshima et al. (2015)

Supplementary Table 3. He isotopic ratios, $\delta^{13}C$ values and $CO_2/^3He$ ratios in high temperature volcanic gases

Volcano	Location	Temperature (˚C)	$^3He/^4He$ (R_a)	$\delta^{13}C$ (‰)	$CO_2/^3He$ (10^9)	Upper mantle	Sediment	Limestone	Reference
Klyuchevskoy	Kamchatka	1100	6.8	-11.6	11.0	20.0%	34.3%	45.7%	Taran et al. (1991); Taran (2009)
Koryak	Kamchatka	220	5.3	-11.8	36.0	6.1%	38.0%	55.9%	Taran (2009); Taran et al. (1997)
Avacha	Kamchatka	473	7.1	-5.7	23.0	9.5%	16.8%	73.7%	Taran (2009); Taran et al. (1997)
Mutnovsky	Kamchatka	543	8.2	-9.9	13.0	16.9%	29.3%	53.8%	Taran (2009); Taran et al. (1992)
Chirpoy	Kuril	344	6.3		59.0	3.7%			Taran (2009)
Kudryavy	Kuril	920	6.8	-7.2	9.0	24.4%	18.7%	56.9%	Taran (2009); Fischer et al. (1998)
Usu	Japan	750	5.4	-4.4	10.0	22.0%	9.9%	68.1%	Marty et al. (1989)
Kuju	Japan	351	7.6	-7.9	13.7	16.0%	22.9%	61.1%	Nagao et al. (1981); Saito et al. (2002)
Unzen	Japan	803	7.3	-6.1	8.1	27.3%	14.2%	58.4%	Sano & Williams (1996)
Satsuma-Iwojima	Japan	885	7.9	-5.5	6.3	34.9%	10.8%	54.3%	Taran (2009); Marty et al. (1989)
Merapi	Indonesia	803	7.4	-3.9	14.0	15.7%	9.6%	74.7%	Taran (2009); Javoy et al. (1986)
Lewotolo	Indonesia	490	3.6	-3.9	12.6	17.5%	9.3%	73.3%	Varekamp et al. (1992)
Ngawha	New Zealand	269	6.0	-8.7	28.3	7.8%	27.2%	65.1%	Giggenbach et al. (1993); Lyon and Hulston (1983)
White Island	New Zealand	495	6.1	-2.0	35.9	6.1%	5.5%	88.4%	Marty & Giggenbach (1990)
Ohaaki	New Zealand	280	5.0		68.5	3.2%			Giggenbach et al. (1993)
Mokai	New Zealand	300	6.8		13.2	16.6%			Giggenbach et al. (1993)
Ngauruhoe	New Zealand	640	6.7	-10.4	7.9	27.7%	28.7%	43.6%	Giggenbach et al. (1993); Giggenbach (1996); Fischer (2008)
Cerro Negro	Nicaragua	350	6.8	-2.5	33.3	6.6%	7.0%	86.4%	Sano & Williams (1996)
Momotombo	Nicaragua	747	7.1	-2.8	16.0	13.7%	6.2%	80.1%	Taran (2009); Javoy et al. (1986)
Pacaya	Guatemala	965	4.2	-6.9	19.7	11.2%	20.7%	68.1%	Sano & Williams (1996)
Galeras	Colombia	222	8.4	-7.6	15.2	14.4%	22.2%	63.3%	Sano & Williams (1996)
Cumbal	Colombia	257	6.8	-4.9	11.7	18.8%	12.3%	68.9%	Sano & Williams (1996)
Colima	Mexico	814	6.6	-6.3	6.1	36.1%	13.2%	50.8%	Taran (2009); Sano & Williams (1996)
La Primavera	Mexico	260	6.2	-3.9	6.2	35.5%	5.3%	59.2%	Taran et al. (2002)
**Average**			**6.5**	**-6.4**	**19.9**	**11.0%**	**18.9%**	**70.1%**	
End-member:									
Upper mantle			8.0	-6.5	2.2	100%	0%	0%	Gerlach (1991); Javoy et al. (1986); Marty & Jambon (1987); Marty & Tolstikhin (1998)
Sediment			0.01	-30.0	10000	0%	100%	0%	Hoefs (1980)
Limestone			0.01	0.0	10000	0%	0%	100%	Hoefs (1980)

Supplementary Table 3
Kagoshima et al. (2015)
